# Supplementary material for: Comparative Analysis of Iodine Levels, Biochemical Responses, and Thyroid Gene Expression in Rats Fed Diets with Kale Biofortified with 5,7-Diiodo-8-Quinolinol
Source: Int J Mol Sci. 2025 Jan 19;26(2):822. doi: 10.3390/ijms26020822 (PMC11765731; doi:10.3390/ijms26020822)
Supplement: Supplementary file 1 [file ijms-26-00822-s001.zip › ijms-3394768-supplementary.pdf]

International Journal of *Molecular Sciences*

MDPI

Supplementary Materials

Comparative Analysis of Iodine Levels, Biochemical Responses, and Thyroid Gene Expression in Rats Fed Diets with Kale Biofortified with 5,7-Diiodo-8-Quinolinol

Table S1. Iodine content in the diet of experimental rats.

Diet type	Iodine content
C	$0.286^{a} \pm 0.001$
CO	$0.276^{a} \pm 0.005$
BO	$0.617^{b} \pm 0.055$
CR	$0.271^{a} \pm 0.012$
BR	$0.531^{b} \pm 0.023$

Diet type: C, control diet (AIN-93G); CO, diet containing control curly kale 'Oldenbor F_1' without biofortified; BO, diet containing biofortified biofortified with 5,7-diI-8-Q curly kale 'Oldenbor F_1', CR diet containing control curly kale 'Redbor F_1'; BR, diet containing biofortified biofortified with 5,7-diI-8-Q curly kale 'Redbor F_1'. Values in rows with different letters (a, b, c) are significantly different, $p \leq 0.05$ (One-way analysis (ANOVA), standard error (n =8).

Table S2. Ingredients of experimental diets' compositions.

Ingredient (g kg^{-1})	C	CO	BO	CR	BR
Corn starch	532.486	524.716	524.896	526.036	525.456
Saccharose	100	100	100	100	100
Casein	200	200	200	200	200
Soybean oil	70	70	70	70	70
Fiber	50	47.02 [a]	50	50	50
Vitamin mix [b]	10	10	10	10	10
Mineral mix [b]	35	35	35[c]	35	35[c]
Choline	2.5	2.5	2.5	2.5	2.5
TBHQ [d]	0.014	0.014	0.014	0.014	0.014
Biofortified kale	-	-	11.2	-	11.1
Control kale	-	10.75	-	9.98	-

C, control diet (AIN-93G); CO, diet containing control curly kale 'Oldenbor F_1'; BO, diet containing biofortified with 5,7-diI-8-Q curly kale 'Oldenbor F_1'; CR, diet containing control curly kale 'Redbor F_1'; BR, diet containing biofortified curly kale 'Redbor F_1'. [a] according to AIN-93G. [b] mineral mix without iodine; in these diets, the source of iodine was biofortified curly kale. [c] tert-butylhydroquinone. [d] freeze-dried curly kale.

 https://doi.org/10.3390/ijms26020822
